# Towards an integrated approach to health and medicine in Africa

**DOI:** 10.1080/17290376.2016.1220323

**Published:** 2016-08-18

**Authors:** Kezia Batisai

**Affiliations:** ^a^ Is a Lecturer in the Department of Sociology, University of Johannesburg, Johannesburg, South Africa

**Keywords:** HIV, AIDS, alternative therapies, health-seeking behaviour, medical humanities, VIH, SIDA, thérapies alternatives, comportement de recours aux soins, sciences humaines médicales

## Abstract

This article frames the intersections of medicine and humanities as intrinsic to understanding the practice of health care in Africa. Central to this manuscript, which draws on empirical findings on the interplay between HIV and AIDS and alternative medicine in Zimbabwe is the realisation that very limited research has been undertaken to examine ‘HIV/AIDS patient behaviour’ with respect to choice of therapy on the continent [Bene, M. & Darkoh, M. B. K. (2014). The Constraints of Antiretroviral Uptake in Rural Areas: The Case of Thamaga and Surrounding Villages, Botswana. SAHARA-J: Journal of Social Aspects of HIV/AIDS, 11(1), 167–177. doi:10.1080/17290376.2014.972057; Chavunduka, G. (1998). Professionalisation of Traditional Medicine in Zimbabwe, Harare, Jongwe Printers; O’Brien, S. & Broom, A. (2014). HIV in (and out of) the Clinic: Biomedicine, Traditional Medicine and Spiritual Healing in Harare. SAHARA-J: Journal of Social Aspects of HIV/AIDS, 11(1), 94–104. doi:10.1080/17290376.2014.938102]. As such, a social approach to health-seeking behaviour questions how decisions about alternative therapies including herbal remedies, traditional healing and faith healing are made. The paper unpacks the realities around how people living with HIV and AIDS – who span different age groups and profess various religious backgrounds, faced with an insurmountable health challenge against a background of limited resources and no cure for the virus – often experience shifts in health-seeking behaviour. Grappling with seemingly simple questions about ‘when, where and how to seek medical attention’, the paper provides pointers to therapy choices and health-seeking behaviour; and it serves as a route into deeper and intense healthcare practice explorations. In conclusion, the paper proposes that medicine and the humanities should engage seriously with those social aspects of HIV and AIDS which call for an integrated approach to healthcare practice in Africa. If combined, medicine and the humanities might achieve what neither would alone.

## Résumé

Cet article présente la convergence entre la médecine et les sciences humaines comme étant un facteur inhérent à la compréhension de la pratique des soins de santé en Afrique. Au cœur du présent article, dont le contenu est fondé sur résultats empiriques portant sur l'interaction entre le VIH/SIDA et la médecine alternative au Zimbabwe, se trouve le constat selon lequel il n’y a eu que très peu de recherches qui ont été menées pour examiner « le comportement des patients atteints du VIH/SIDA » en ce qui concerne le choix de la thérapie sur le continent [Bene, M. & Darkoh, M. B. K. (2014). The Constraints of Antiretroviral Uptake in Rural Areas: The Case of Thamaga and Surrounding Villages, Botswana. SAHARA-J: Journal of Social Aspects of HIV/AIDS, 11(1), 167–177. doi:10.1080/17290376.2014.972057; Chavunduka, G. (1998). Professionalisation of Traditional Medicine in Zimbabwe, Harare, Jongwe Printers; O’Brien, S. & Broom, A. (2014). HIV in (and out of) the Clinic: Biomedicine, Traditional Medicine and Spiritual Healing in Harare. SAHARA-J: Journal of Social Aspects of HIV/AIDS, 11(1), 94–104. doi:10.1080/17290376.2014.938102]. A ce titre, une approche sociale au comportement de recours aux soins de santé remet en cause la manière dont sont prises les décisions dans le cadre des thérapies alternatives, y compris les remèdes à base de plantes, la guérison traditionnelle et la guérison par la foi. L'article révèle les réalités autour de la manière dont les personnes vivant avec le VIH/SIDA – de différents groupes d'âge et de diverses confessions religieuses ayant connu des défis sanitaires insurmontables dans un contexte marqué par les ressources limitées et l'inexistence de remède pour le virus – connaissent souvent le changement comportemental lié à la recherche de soins de santé. Aux prises avec des questions apparemment simples sur « le moment, l'endroit et la façon de recourir aux soins médicaux », l'article fournit des éléments indicatifs sur les choix de la thérapie et le comportement de recours aux soins; il sert également de guide vers l’examen intense et profond de la pratique des soins de santé. En conclusion, l’article recommande que la médecine et les sciences humaines se penchent sérieusement sur les aspects sociaux du VIH et du SIDA qui exigent une approche intégrée de la pratique des soins de santé en Afrique. La médecine et les sciences humaines peuvent réaliser ensemble ce que chacune d'elles ne pourrait pas accomplir toute seule.

## 1. Introduction

Global-wide scholarship tracks down the history of medicine and humanities demonstrating how discourses in these fields have evolved over the years. Although the work of Cassell (as cited in Scott 2000:4) asserts that ‘the humanities have always been part of medicine’, a historical mapping of literature suggests otherwise. Long-standing constructions of humanities and medicine as two separate disciplines are documented by Fieschi, Matarese, Vellone, Alvaro and De Marinis (2013); and Aull (2012) who among other scholars (Abdel-Halim & Alkattan 2012; DasGupta 2003; Humayun & Herbert 2011; Powley & Higson 2013) also illuminate their subsequent integration within specific contexts across the globe. In America, the movement for an integrated approach to medicine and humanities in the 1950s–1960s was informed by anthropological medical research developments specifically on interpretations of illness and treatment along with efforts towards a human-centred approach to care (Fieschi *et al*. 2013:56). The relationship between the arts and medicine in Europe and America is analysed by Scott (2000) who draws on existing scholarship to expose and justify the contribution humanities makes to medicine. Cassell (as cited in Scott 2000:4), for instance, acknowledges that if integrated with humanities, medicine will shift from its current interest in technology ‘towards a more balanced view of the origin and treatment of illness’. In South Africa, an interdisciplinary approach to medicine and humanities strives to ensure that health science students are alert to patient autonomy and celebrate cultural difference in ways that allow them to respect varied interpretations and meanings attached to ‘the illness experience’ (Reid 2014:109–110). Particular attention is on conceptualisations of health and disease from a sociological perspective deeply embedded in a discussion on emerging discourses on medicine and humanities on the continent.

The need to engage with the interplay between medicine and humanities is evident in analyses of what health and disease are, which do not limit conceptualisations of such to purely biomedical definitions. These conceptualisations take into account the social constructedness of health and disease which encompass contextual meanings, health-seeking behaviour, gender differences, and sociocultural, economic and political terrains, among other factors. The work of Pretorius (2014:385) reiterates the micro and macro impact the social environment has on health and disease originally captured in the 1998 World Health Organisation commissioned report: *Social determinants of health – the solid facts*. Pretorius also concurs with Reid (2014:110) who argues that contextualised analyses of medical humanities take into account cultural, historic, geographical and political factors allowing one to unpack ‘notions of community, traditional forms of healing, or poverty and social justice’. The realisation that social interpretations assigned to ‘being poor, unemployed, socially excluded and stigmatised’ are of great significance to conceptualisations of health and disease forms the very basis for a biomedical framework that draws on sociology (Pretorius 2014:385) among other disciplines like education (Fieschi *et al*. 2013). What sociology is trying to do is not to discredit biomedicine, and its discoveries. Rather, the social model offers particularly interesting and valid criticisms that are ‘complementary to and convergent with medical scientific practice’ (Pretorius 2014:383). Instead of limiting the definition of health to the absence of disease, the conceptualisation of health in the ambit of sociology includes a sense of well-being which simultaneously reflects the sociocultural, economic and political order of any given setting.

The sociological perspective presented above explicitly points at how medicine and humanities intersect – an interplay that experts in both disciplines have to engage. In South Africa and Africa at large, the interplay was and is being explored by intellectuals, medical practitioners and artists who through a conference themed ‘Medical Humanities in Africa 2014’ created a platform that allowed dialogue between medicine and humanities. Central to the Medical Humanities in Africa 2014 were four interdisciplinary themes: *Paradigms, Pedagogies, Practices* and *Potential*. It is noteworthy that *Practices* was founded on a couple of assertions and questions fundamental to the arguments explored in this article. For instance, *Practices* set to establish ‘the contribution humanities make to health care practice in Africa and the direct relevance of humanities to health professionals’. As previously alluded to (Pretorius 2014; Reid 2014), the significance of sociology among other humanities is clearly discernible in the use of alternative therapies and traditional healing in medicine.

### 1.1. Motivation for the study

Medical Humanities in Africa 2014’s interest in ‘research results and reflections on practice, or the implications of new ways of seeing on the practice of healthcare’ opened space for re-engagement with unpublished findings from my previous research (Batisai 2004) which explored the intersections of HIV and AIDS, alternative medicine and health policy recommendations in Zimbabwe. Although one might question the relevance of data that were collected more than a decade ago, the findings from this fieldwork are profound for they speak to very recent scholarship (Bene & Darkoh 2014; O’Brien & Broom 2014; Olaore & Olaore 2014; Olowu 2015) on therapy-seeking behaviour, HIV and AIDS, and responses to the pandemic in Botswana, Zimbabwe, Nigeria and Lesotho, respectively. Discourses from these contexts not only concur with patterns of health-seeking behaviour of people living with HIV and AIDS (PLWHA) that emerged out of my research in Zimbabwe. The scholarship offers theoretical and empirical evidence that somewhat legitimises the main proposition of this manuscript: towards an integrated approach to health and medicine in Africa. Thus, the need to provide more insight into ‘how empirical and analytical results can be translated into actual changes in the way that health care is offered’ (Medical Humanities in Africa 2014) is central to discussions engaged in this article.

Subsequent to the foundation laid above, the next section of the paper maps the healthcare landscape in Africa profiling the realities around how traditional and biomedicine have been separated or integrated in contexts such as South Africa and Zimbabwe. The African-focused discussion grapples with fundamental questions: What story does the integration or separation of traditional and western medicine tell us about health care, humanities and medicine in Africa?; what impact does the integration or separation of the two have on health-seeking behaviour, especially among PLWHA?; and what overall narrative do we get about health and medicine in Africa?

## 2. Literature review

Developing contexts such as Africa are characterised by a long-standing ‘traditional and prescientific’ healthcare system often juxtaposed with another system framed as ‘modern, scientific and Western in derivation’ (Good, Hunter, Katz & Katz 1979:141). Shale, Stirk and van Staden (1999:347) trace the history of medicine and note that plants have a long history of providing ‘mankind with a source of medicinal agents’ (see also Sindinga, Nyangiotti-Chacha & Kanunah 1995). A significant number of people located in urban (Pretorius 1991) and rural areas (Shale *et al*. 1999; Truter 2007; Umar, Rabah, Na’ala, Bello, Ibrahim & Garba 2011) of developing countries across the globe rely on traditional medicine or remedies (Good *et al*. 1979). For instance, Shale *et al*. (1999:347–348) reveal that 80% of the rural population in Africa among other developing contexts uses traditional remedies (see also Truter 2007). Concurring with such conclusion, Umar *et al*. (2011:6473) draw on findings from rural Nigeria where over 60% rely on traditional remedies. Similarly, about 60% to 80% of the South African population explores traditional healing prior to accessing primary health care (Kale 1995:1182; Truter 2007:56).

In their analysis of the wide use of traditional medicine in rural areas, Umar *et al*. (2011) argue that these remedies are often readily available, affordable, and cheaper than Western medicine since they are ‘mostly compounded from natural products’. Empirical evidence in Gelfand’s 1985 and Chavunduka’s 1994 researches (as cited in Gudhlanga & Makaudze 2012:75) suggests that ‘about 234 of the plants said to be of medical value in Zimbabwe were also used for the treatment of medical complaints in Central, East and West Africa’ and over a quarter of these plants were used for similar ailments across the regions. The statistics above clearly expose why traditional medicine is framed as a reality for both rural and urban contexts (Pretorius 1991:10) especially when access to mainstream health care is limited as case studies from Botswana (Bene & Darkoh 2014); Zimbabwe (O’Brien & Broom 2014); Mozambique (Jaiantilal, Gutin, Cummings, Mbofana & Rose 2015); Kenya, South Africa and Zambia (*SAfAids News* 2004) suggest.

Research findings from Morrumbala Centre, Zambezia Province in Mozambique reveal that ‘a majority of chronically ill patients interviewed stated that they had sort treatment from traditional healers at some point’ (Breslin & D’Allesandro 2004:5). In South Africa, economically challenged citizens also rely on a wide range of healthcare systems including western, faith healing and traditional (Kautzky & Tollman 2008:27). The fact that it is every citizen’s right to hold and belong to any religious group in South Africa (Van Niekerk 2011) somewhat institutionalises religion and gives individuals the freedom of choice which, in the context of health-seeking behaviour, manifests through opting for faith healing.

That notwithstanding, there are challenges around the use of traditional medicine on the continent. Very little has been done to ensure the integration of traditional and western health systems (Pretorius 2014:383). This separation is captured by Shale *et al*. (1999:353) who point at limited collaboration and unidirectional interaction between traditional and western doctors evident in the way traditional healers and herbalists sometimes refer patients to Western doctors without any reciprocal action from the latter. For that reason, traditional healers in Mozambique pushed for collaborative work with the Department of Health as part of their effort ‘to improve care for the chronically ill’ (Breslin & D’Allesandro 2004:5).

The separation alluded to above could be explained by the stigmatisation of traditional medicine faced in light of modern/primitive dichotomies (Good *et al*. 1979) quite dominant in the early days of colonialism (Pretorius 2014:405) and the impact of increased westernisation in subsequent years (Chavunduka & Last 1986). Combined, these factors did not deter Africans from utilising traditional medicine (Gudhlanga & Makaudze 2012) such that the use of traditional medicine post-independence became one of the ways Africans deployed to ‘rediscover their sociocultural identity’, and to deal with inaccessible and expensive medicines foreign to them (Pretorius 1991:10). For Pretorius, such perseverance, along with the economic challenges impelled the powers that be to explore traditional medicine and the merits of having a ‘syncretic national health care delivery system’ in Africa (10).

Integration in Zimbabwe could be traced back to 1980 when the government legitimised traditional medicine with the formation of Zimbabwe National Traditional Healers Association as the country emerged from colonial rule (Chavunduka 1998). Although this formalisation resulted in collaborative work between traditional healers and the Ministry of Health and Child Welfare, the ‘marriage’ was short-lived because the Association felt exploited and withdrew from the arrangement. Nevertheless, traditional remedies are readily available in pharmacies and supermarkets across the country (O’Brien & Broom 2014). In South Africa, ‘there has been a significant degree of legitimation and acceptance of complementary and alternative medicine by biomedicine’ (Pretorius 2014:383) to the extent that the primary healthcare movement in the country adopts a ‘holistic approach which has taken an interest in traditional healing systems’ (404). Despite the legal challenges (Gudhlanga & Makaudze 2012; Truter 2007), traditional medicine continues to flourish in many African countries (Kale 1995), where mainstream systems have historically failed to meet healthcare demands of the public (Good *et al*. 1979). Hence, the belief that if incorporated into health care ‘traditional healing […] could improve the way of life for many people, especially those in rural areas’ (Shale *et al*. 1999:353) who often do not have money for transport costs to and from healthcare centres (Bene & Darkoh 2014; Kautzky & Tollman 2008; O’Brien & Broom 2014).

The realities noted above could be the reason behind fairly recent global focus on ‘the development of medicinal plants and traditional medicine’ (Umar *et al*. 2011:6473). For instance, empirical findings from Lesotho profile a wide range of plants with medical value, how they are processed, and the subsequent utilisation of such (Shale *et al*. 1999). Although very little has been done to explore the merits of *Celosia leptostachya* in Nigeria, it emerged from Umar *et al*.’s (2011:6474, 6476) study that the plant has medicinal powers and it could serve as an ‘alternative source of antibiotics’. Overall, scholarship on Africa’s healthcare landscape and alternative healing practices prepares this article to engage with contextual analyses of health and medicine largely informed by empirical findings on health-seeking behaviour from Harare, the capital city of Zimbabwe. The analytic discussion concludes by identifying gaps calling for further action and making recommendations for the recognition, support and regulation of alternative therapies – meaningful to the way health care is offered in Africa.

## 3. Methodology

Before fieldwork commenced in Harare at an organisation run for and by PLWHA, I sought permission from the authorities at this organisation in order to gain entrée. It is worth mentioning that I selected the organisation and the interviewees for this study using purposive sampling – a process that allows researchers to consciously choose a research setting and participants who are perceived to be rich sources of information about the subject under study (Miller 2000:78). With the help of staff at the organisation, I managed to gain entrée and I purposively selected 25 participants (both men and women) based on their willingness to share their experiences and to be interviewed in depth on several occasions. In order to increase interaction levels and bridge the outsider/insider gap, the staff at the organisation helped me to participate in a behaviour change programme and I was assimilated into the group. Participation was instrumental because it emerged that PLWHA are referred to as clients and not patients to reduce stigmatisation.

Fieldwork was designed in a way that allowed one to establish: how and why PLWHA from different parts of Harare made therapy choices; types of healing therapies pursued; utilisation patterns of different therapies that emerged out of their narratives of health-seeking behaviour; and issues (side effects, if any) associated with the therapy selected. I adopted a qualitative methodology because of its great ability to locate the researcher in the social setting where interaction takes place offering deeper insight into complex realities (Neuman 1997). One-on-one in-depth interviews created room for me to build an intimate relationship with all 25 participants, and space for gaining trust that allowed me (together with the interviewees) to further confront and capture the realities of living with HIV/AIDS through follow-up interviews. I designed an interview schedule which outlined flexibly worded themes (noted above) and questions to be covered, and this schedule helped me to collect comprehensive data in a methodical way. Interview sessions were fairly conversational and situational (Ulin, Robinson, Tolley & McNeill 2002:64) allowing lived realities to be conveyed in participants’ own words. In addition, I had informal interviews with key informants who sourced a wide range of information on herbal remedies in Zimbabwe. The conversational and situational approach alluded to above allowed me to rely on note-taking in the absence of any audio or video recording devices. I was the only person that had access to the journal with the interview notes which I destroyed when the study ended.

Drawing on the notion of data triangulation in qualitative research (van Rensburg 2010), I also reviewed secondary data sources. Secondary data emerged extremely resourceful because academic and non-academic articles set the analytic framework, theoretical arguments and contextual parameters guiding this manuscript. Data are therefore presented in a way that integrates participants’ narratives with theoretical and empirical findings from other African contexts profiled in existing scholarship. The analytic framework clearly demonstrates how significant the literature (reviewed) is to the findings from Harare, and the study at large. Even though 25 PLWHA were involved in this study, the findings section only works with 10 (40%) of these participants (seven women and three men) whose narratives explicitly speak to the central questions addressed in this manuscript.

### 3.1. Ethical considerations

Prior to fieldwork, ethical approval was granted by the Ethics Committee at my university, and a formal consent letter was endorsed by my academic department. Alert to the major ethical issues around the need to ‘protect, respect and be accountable to’ the people involved in the research processes (Bennett 2008:5; Clark & Sharf 2007:400), I ensured that all 25 participants were informed about the nature and objectives of the study beforehand. Within this understanding, there was a notion of proper respect of human freedom such that all 25 agreed voluntarily to participate without any fear of deception and physical or psychological harm. To conform to the ethics around individual privacy and confidentiality, all identifying data are concealed, and participants’ narratives are only published under the shield of anonymity. Any personal biographical information peripheral to the research is not presented in this manuscript to avoid making ‘the personal’ public (Miller 2000:82).

## 4. Findings

As noted earlier, a total of 25 HIV positive people, who span different age groups (between 19 and 46 years) and profess various religious backgrounds, participated in this study. Out of these 25 participants, 88% were from the low-income group while only 12% were from the middle to upper class. Furthermore, 20 of the total participants were women (80%) and only 5 (20%) were men. It emerged in this study that although both men and women seek alternative medicine including herbal remedies, women tend to outnumber men. The reason for this could be that as wives, women stick to long-standing beliefs, especially gendered traditional and cultural values, which make it difficult for women to negotiate safe sex and put them at risk of being infected (Jaiantilal *et al*. 2015; Njovana & Watts 1996). Moreover, their role as traditional caregivers of the sick which intensified in the context of HIV/AIDS and home-based care puts them at risk of infection (Jackson 2002; Siliwa 2007). As well as being more vulnerable to the virus, women are also more open about their HIV status and thus are more likely to seek assistance compared to men (Jaiantilal *et al*. 2015). Furthermore, many African women also take herbs during pregnancies (Gudhlanga & Makaudze 2012) and hence are more prepared to accept herbal medicine than men. The analyses above speak to the power of sociological interpretations of health and disease, especially their acknowledgement of the impact of social factors such as ‘social power, religious views and even gender and ethnicity’ in making sense of health, disease, and the subsequent health-seeking behaviour (Pretorius 2014:386; Reid 2014). That questions such as ‘why women are more frequently diagnosed ill than men require a sociological explanation rather than a biological one’ (Pretorius 2014:386) further illuminate the value of these interpretations.

The research revealed that there are different types of herbs that are and can be utilised by PLWHA. These include local and imported herbs such as aegle mermalos/wood apples, alfalfa/lucerne, aloe vera/*gavakava*, amaranth/*mowa guru*, anise**,** basil, eucalyptus/gum, comfrey, garlic, parsley, ginger, impi, rosemary, sage, St John’s wort, and sutherlandia. Scholarship reveals that herbal remedies are prepared and administered in different ways (see Kale 1995; Shale *et al*. 1999; Umar *et al*. 2011) but often they are an infusion of the whole herb or its parts such as flowers, seeds, leaves, roots or bark (Gudhlanga & Makaudze 2012; SAfAids News 2004; Shale *et al*. 1999; Umar *et al*. 2011). The participants used different herbal remedies to manage several opportunistic infections and conditions including constipation, diarrhoeal infection, symptoms of inflammation, viral and fungal infection, spasmodic coughs, sore throat, oral thrush, high cholesterol and blood sugar levels, anaemia, scalp infection, skin ulcers, pneumonia, migraines, depression, frigidity, menstrual problems, loss of energy, appetite and body weight; Candida or thrush and sexually transmitted infections such as syphilis and gonorrhoea. Some of these conditions concur with those documented in the works of Gudhlanga and Makaudze; SAfAids News; Shale *et al*. and Umar *et al*. cited above.

### 4.1. Why PLWHA used alternative remedies

Often, people have a choice in terms of when and where to seek therapy, whether one should use western medicine, traditional medicine or faith healing (Chavunduka 1985:10). In line with Weber’s instrumental rationality (Campbell 1981:176; Johnson 1981), participants assessed the best therapy system through the process of gathering information, taking note of opportunities and obstacles in the environment, and attempting to predict the possible consequences of an alternative line of action. The choice of herbal remedies and their acceptance by the majority of PLWHA was based on various factors and the primary ones being inaccessibility and unaffordability of western medicine coupled with their strong belief in herbs as effective, affordable and accessible remedies. This concurs with the argument that herbal remedies are readily available (especially in rural areas), affordable and cheaper compared to Western medicine, an inference located in the fact that such remedies are ‘mostly compounded from natural products’ (Umar *et al*. 2011:6473) as noted earlier by Anyiman (1987).

By the turn of the twenty-first century, poor HIV-positive people could not easily access anti-retrovirals (ARVs) in Zimbabwe (O’Brien & Broom 2014), and it cost a patient an approximate of US$1052 a month to purchase privately imported drugs (Mutetwa 2001). Besides the cost, the fact that ARVs should be taken for the rest of one’s life alongside a balanced diet made it very difficult for low-income PLWHA located in Zimbabwe – a country that has experienced drastic socio-economic and political shifts^1^ (Bond & Manyanya 2003) – to utilise these tablets. In a context where PLWHA had limited income, herbal remedies became the most rational alternative therapy route, which, beyond controlling the illness, provided the body with nutrients required in times of sickness to speed up recovery. Therefore, PLWHA as revealed below rationally took into account and weighed both the means and ends to determine a cost-effective alternative, which brought hope to them in a bleak environment.

For some clients who still have close ties with their rural homes they can get the local herbs from the forests. (A 46-year-old HIV positive male participant)

Clients get their herbs and also seedlings to establish their own herbal gardens at home from the garden at TC. We established a herbal garden at our backyard which makes herbal remedies available and accessible at almost zero cost. This is also a good idea for PLWHA in an urban setting since it reduces the cost of going to the organisation every month to collect herbs. This whole process makes local remedies simple, easily accessible and readily available, and enables clients to be self-reliant. Clients end up mixing their own remedies from medicinal plants grown at their homes or the organisation to keep the costs down. (A 37-year-old HIV positive female participant)

The cost of western medicine in most developing countries, Zimbabwe included, is often beyond the reach of poor citizens such that its services only reach a few who can afford; and even where public health facilities exist, medical service is not always available (Good *et al*. 1979; Kautzky & Tollman 2008; Truter 2007). Many healthcare centres on the continent are ill equipped with respect to health personnel, supplies of drugs, transport problems, water and sanitation, stationery, and overall management and infrastructural maintenance of these facilities. In the context of South Africa, there is ‘unequal distribution of human resources between the private and public sectors’ (Pretorius 2014:404), a challenge rooted in the apartheid system (Kautzky & Tollman 2008:20). All these problems translate into non-availability of services. Houston (2001) therefore argues that any healthcare system, which provides a service that is neither affordable nor accessible, can never claim to be effective and efficient. As such, the majority of participants from the low-income bracket noted inaccessibility of western medicine as the main reason behind the choice to make use of herbal remedies.

I have heard of ARVs but I don’t have the money to buy them so I make do with what I have. (A 46-year-old HIV positive female participant)

The decision was made on my behalf in 2001 by our sahwira [a family friend] after my family had deserted me. It also came as a desperate measure following the fact that I was unable to earn a living … I could not even afford … I had no money for hospital fees. Despite the wonderful work of these remedies, I am a sick person because my immune system is now compromised although my body appears healthy. (A 46-year-old HIV positive male participant)

It is noteworthy that there were a few participants (12% of the 25 participants) from either middle or upper class and their choice of herbal remedies was not because they could not afford western medicine. Rather, herbal remedies were a last resort or desperate measure after the western medical approach had failed them. This category of people became desperate due to the fact that there is no cure and when western medicine failed them, PLWHA tried whatever therapies that promised relief and the restoration of health ranging from faith healing to herbal remedies. It also emerged that those in the middle and upper income groups constituted what is referred to as ‘walk in clients’ who just visited the organisation for counselling services and preferred to purchase their herbs over the counter. Although few, these middle–upper class cases concur with Pretorius (2014:386) who identifies ‘socio-economic class’ as one of the factors that impact not only on medical knowledge but on the ultimate health-seeking decision and behaviour.

Good (1982) notes that the definition of illness differs from society to society. Many African communities categorise disease and illness according to cause. There are some illnesses that are believed to be caused by social agents such as ancestral spirits, angered spirits and witches (O’Brien & Broom 2014; Truter 2007). Illness is *chirwere chavanhu* (human induced) and *chirwere chaMwari* (naturalistic) when it is believed to have been caused by witches and spirit disturbances with regard to ancestors and God-given, respectively (Mararike 1999:111). Health and disease in such contexts are perceived as ‘social constructs or “products” of social organisation rather than of nature, biology or even individual lifestyle choices’ (Pretorius 2014:386). In South Africa, between 60% and 80% of the population relies on traditional medicine predominantly because of their deep-rooted cultural and traditional beliefs, which associate sickness and disease with the spiritual kingdom (Truter 2007:56–59). Where an illness is believed to have been caused by these social agents, PLWHA, and people in general, find it rational to use alternative remedies only since they believe that western medicine is unable to handle such illness. Pretorius (2014:386) points at how central ‘social factors and the prevailing societal influences’ are to the process of conceptualising and assigning meaning to health and disease (see also Reid 2014). Hence, the need to attend to the contribution humanities makes to medicine summarised below,

Based on their research, sociologists of health and disease are able to demonstrate how the development of medical knowledge and the treatment of disease are powerfully influenced by social factors […]. Sociologists are […] able to provide information on many aspects of disease causation other than biological variables – on patterns and trends pertaining to various diseases, on the practice of medicine and on people’s perceptions and experiences of and also their responses to being ill. (Pretorius 2014:386)

In each and every society, there are norms and values, which are regulating and directing principles, and in the absence of these norms, individuals are left without moral guidance in the pursuit of their goals (Coser 1981:133). The *runyoka* phenomenon (fencing off women), a cultural belief held by the Korekore people of Zimbabwe as indicated by *a 22-year-old HIV-positive male participant,* appears to be part of the social and moral control on family and lineage life. It governs a wide array of aspects of everyday activities ranging from sexual property to communal life. The Korekore believe that breaching taboos, especially sexual ones, results in illness and conditions said to be incurable through western medicine (O’Brien & Broom 2014). This in turn influences therapy-seeking and selecting behaviour since many communities categorise disease and illness according to cause. Following Weber’s instrumental rationality, PLWHA therefore tend to rationally take into account the means and the ends of alternative medicine avoiding lines of action which clash with their cultural or religious beliefs as evidenced by the narratives below.

We believe that western medicine is unable to handle illnesses caused by people such as runyoka and the traditional healer is the only person who can reverse the situation. (A 22-year-old HIV positive male participant)

I am a devoted member of the Jowani Masowe eChishanhu an Apostolic Church which does not allow its members to use herbal remedies or even go to hospital. In times of sickness, members only drink and bath in holy water and oil, and eat raw eggs blessed by senior prophets irrespective of one’s condition. (A 37-year-old HIV positive female participant)

It is significant to note as Janzen (1978) found among the Bakongo of lower Zaire that the decision to seek therapy and where to seek it does not lie with the patient alone, but it is made by the therapy-managing group comprising family members and close friends. Almost 10 participants indicated that the decision to seek therapy in herbal remedies was made by self, family members (father, husband, wife, aunt) or friend. For example, the 46-year-old HIV positive male participant’s choice and decision was initially made by his wife and later on by a friend. The therapy-managing group made judgement on the efficiency and effectiveness of the use of herbal remedies as an alternative line of action as highlighted by Weber’s instrumental rationality. The therapy-managing group also accumulated knowledge and experience passed on from generation to generation through the process of socialisation. This is illustrated below by the excerpts of participants who learnt about traditional remedies from senior family members.

As a daughter of a famous traditional healer, my father educated me on the advantages of herbal remedies and how to mix herbs to treat sexually transmitted infections. He told me that since the remedies are in their natural state they have several helpful ingredients. In 1999, my husband was involved in a car accident and lost a lot of blood. As a wife, I offered to donate some blood to save my husband’s life. This was the time when my HIV status was discovered. The doctor, who was an army medic had a strong passion for natural remedies, referred me to the organisation. I was prepared to take the doctor’s advice because I had seen these remedies work very well on my father’s clients who had sexually transmitted infections and I used to admire the speed recovery. Currently, I am using herbal remedies and in order for these remedies to work, one has to first accept and have faith in them because for any medicine to be effective it all starts in the mind. (A 31-year-old HIV positive female participant)

My grandmother used to go into the forest to access some plants that were believed to have medicinal powers. So when I first contracted a sexually transmitted disease in 1991 I went back to my grandmother for some herbs to stop the diarrhoea and they worked very well. But as you know, old habits die hard; I continued to engage in commercial sex work with soldiers who had lots of disposable cash especially after the war in Congo [DRC]. By 2002, I was diagnosed HIV positive after a relative who works at a Voluntary, Counselling and Testing centre had given me some advice. I was then referred to the organisation where I was given some herbs. I chose to use herbal remedies because this is what I grew up using. I am so proud of these herbs so much that I recommended them to my sick friends who used to engage in commercial sex work like me. (A 40-year-old HIV positive female participant)

These participants’ experiences reinforce the sociological theorisation that

everything we know about health and disease or of the professions that deal with them or how we respond to and experience being healthy or ill are moulded by the society in which we live and our place in that society. (Pretorius 2014:386)

Similar to the findings from Botswana (Bene & Darkoh 2014), the process of gathering information about choice of therapy for the 10 participants depended on factors such as the cost of each type of treatment and its accessibility. In addition, the decision was also informed by their knowledge of the probable effects of each kind of treatment and the contextual definition given to that particular illness by the participant and members of his or her social group.

### 4.2. Utilisation patterns

A close examination of health-seeking behaviour among participants reveals that herbal remedies and western medicine can interact in different forms. One of these forms of interaction has been defined by Romanucci (1999) as sequential zigzag (see also Pretorius 1991). In this form of relationship, a client may start using either of the two forms and moves on to the other and then returns to the form that he/she had used initially. This is well demonstrated by the 46-year-old HIV-positive female participant who first used remedies and moved on to western medicine but when western medicine failed her due to inaccessibility, unaffordability and unavailability issues, she returned to remedies. Thus, as the illness develops, there is oscillation between the two types of medicine often caused by dissatisfaction with a specific therapy choice. The way a 19-year-old HIV-positive female participant (see page 7) also moved freely between the organisation and the hospital clearly indicates that choice of therapy is fluid. When the 27-year-old HIV-positive female participant in the excerpt below developed some symptoms, her first choice of therapy was western medicine, but she did not obtain a cure so she turned to remedies.

In 2002, I experienced serious diarrhoea, chest pains and severe bouts of both vaginal and mouth thrush and I gave first preference to western medicine. After being diagnosed and with a very low CD4 count, the doctor gave me a prescription which indicated that I should try one of the leading AIDS drugs, AZT. I did not feel the expense because a friend based in the United Kingdom used to send me money to buy the drug. Due to the drug’s side-effects, I started to experience dizziness, constant toilet visits and loss of appetite and weight. After such dissatisfaction, another friend advised me to visit the organisation for herbal remedies where I received some training on how to make use of herbs and their advantages over western medicine. (A 27-year-old HIV-positive male participant)

It is however imperative to note that some patients expect instantaneous, almost miraculous recovery, and they do not seem to wait long enough to complete a course of therapy in a health facility before taking the same episode of illness to herbal remedies. As highlighted by Sindinga (1992:68), this movement from one medical system to another even on the same episode of illness also appears to be influenced partly by the confusion caused by the diffuse nature of symptoms as well as the difficulties of interpreting symptoms and determining the cause of a given illness.

Similar to findings from Botswana (Bene & Darkoh 2014) and Zimbabwe (O’Brien & Broom 2014), men and women who participated in this research often utilised alternative remedies and western medicine concurrently for the same episode of illness. This is common in contexts where there is medical pluralism, which is the existence in a single society of differently designed and conceived medical systems (Janzen 1978:xviii; Pretorius 1991). Such systems exist together and many compete with one another. Medical pluralism is significant in Zimbabwe, especially in the area of decision-making with regard to seeking and selecting therapy among PLWHA. The experience captured below demonstrates that many participants did not stop using western drugs as they begun to use the herbal remedies,

I discovered my HIV status when I visited a VCT centre in 1999. During that period I had meningitis and recurrent pneumonia. As a daughter of a prominent businessman, I was taken to several expensive hospitals in town and even across the border because my father could afford the hospital bills. All this was done without any remarkable results and I suggested to my father to try faith healing and he agreed. I became a full member of a certain Pentecostal church where I was given holy water, milk and raw eggs to treat the meningitis and pneumonia but again, all was in vain. In September 2000, my father came across an article: ‘Traditional medicine: an ally for care. HOPE IS VITAL’ which had details about herbal remedies. My father then took me to an organisation working with herbs and by the end of 2000, my condition had changed. I am now on a regimen of natural medication and a special diet plan from the organisation. I am also using western medicine … ARVs … cotrimoxazole costs me ZW$30 000 and fluconazole ZW$40 000 for four tablets per week. (A 19-year-old HIV-positive female participant)

Commenting on medical pluralism, many participants suggested that such behaviour is probably motivated by the desire to maximise chances of regaining health, especially in situations where they consider both types of medicine as necessary for healing to occur. Responding to the question why she combined western medicine, faith healing, and herbal remedies, the 19-year-old HIV-positive female participant above said, ‘I am trying to utilise both approaches in order to maximise chances of regaining health since there is no cure for HIV/AIDS.’ Arguing that her family made a rational decision by combining these types of medicine, the participant proudly said, ‘I am now well enough to go university or college.’ Similarly, these arguments are relayed by 24-year-old HIV-positive male participant,

I can now afford to relax with others without fear of scratching my private parts when feeling some burning sensations … because the sensations are now under control. (A 24-year-old HIV-positive male participant)

The 19-year-old HIV-positive female participant’s experience also illuminates how PLWHA use various medical systems in a complementary way, which indicates another form of relationship between the two medical systems (Kautzky & Tollman 2008; Pretorius 1991). This relationship exists in cases where only one of the two types of medicine is used in addition to the other for the management of a condition. For example, a 28-year-old HIV-positive female participant heavily relied on herbal remedies along a healthy diet (fruits, vegetables, green leaves) to improve and manage her vitamin deficiency and she only took multi-vitamin tablets to supplement herbal remedies. It is clearly discernible that health-seeking behaviour among PLWHA and patterns that emerge out of utilisation of and relying on different forms of therapy are indeed informed by constitutions that go beyond the individual to encompass social beliefs and environments (Bene & Darkoh 2014; O’Brien & Broom 2014) that vary from context to context, with parallels in between. These social constitutions are further reinforced by the observation that people still rush for herbal medicine in contexts like Zimbabwe where ARVs are given for free in government hospitals. One can therefore conclude that African people, despite long contact with western medicine, still believe and have a lot of faith in traditional medicines.

Despite the limitation that the findings presented in this paper emerged from a study that confined the sample to a single research setting (an organisation in Harare), it is evident that the thematic discussions in this paper speak to the realities of the use of alternative medicines in many African contexts. The limitation also creates room for subsequent research and future publications on the same phenomenon but with a specific focus on people in rural areas. Broadening the research setting and scope allows one to explore the responses why people in rural areas resort to alternative medicine compared to the reasons provided by those in an urban centre of Harare.

## 5. Discussion and conclusion

Scholarship engaged in this manuscript has illuminated that a significant number of the population in many African contexts relies on traditional herbal remedies. It is, however, imperative to acknowledge that traditional healing among many blacks is not only utilised ‘because of limited other choices, but also because they deem it acceptable and functional’ (Pretorius 2014:405). A society which, in the context of HIV and AIDS among other conditions, constructs what it considers to be rational in a way that allows traditional and western medical systems to coexist pushes one to seriously consider an integrated approach to health and medicine in Africa. This conclusion/recommendation is not blindly arrived at. It is cognisant of the fact that

the World Health Organisation […] lent its stamp of respectability to traditional medicine, urging Third World governments not to rely exclusively on western type medicine in attempting to provide health care for all their people but aim for a synthesis between the best of western and the best of traditional medicine. (Melrose 1982:118)

Such synthesis translates into the establishment of a healthcare system that capitalises on the strengths of western and traditional/alternative medical approaches (Good *et al*. 1979; Gudhlanga & Makaudze 2012; Kautzky & Tollman 2008). Integrated, these two systems might be complementary in their merits and flaws and achieve what neither would alone.

The call for integration is partly embedded in the challenges that stem from unregulated use of herbal remedies. Although anyone who has faith in traditional medicine believes that the herbs are not harmful because they are used in their natural state, Akerele (1987:178) argues that as with western medicine there are, of course, both positive and negative sides to herbal remedies. As such, drawing on Gono’s work, Gudhlanga and Makaudze (2012:77) illuminate that medical authorities in Zimbabwe acknowledge the nutritional value and safety of *moringa* leaves and pods simultaneously discouraging the use of toxic and harmful roots of the same tree by patients. Based on the realities that herbal medicines have potential side effects (Kale 1995; Pretorius 2014; Truter 2007) and that they are central to individual and sociocultural identities (O’Brien & Broom 2014; Pretorius 1991) of people who utilise them on the continent, this paper provokes those in the medical fraternity to strive to control the toxicity of remedies to a point where they are safe for human consumption (Shale *et al*. 1999; Umar *et al*. 2011).

Drawing on Elling who poses a fundamental question around how integration ‘will be done and what it will mean’, Pretorius (1991:10) captures the advantages and disadvantages of an integrated approach to health and medicine. Good *et al*. (1979) propose herbal medicine research and referral systems among other strategies. The strategy is supported by Gudhlanga and Makaudze (2012) who encourage all Biochemistry departments of academic institutes across Zimbabwe to invest in research on traditional medicine; and the establishment of a Department of Traditional Medicine at the country’s leading university. A harmonised existence of traditional and western medical approaches could be of great significance to healthcare intervention programmes since ‘there is potential to improve service coverage and delivery and arguably achieve increasingly comprehensive care in a way that is more socially and culturally appropriate to the population’ (Kautzky & Tollman 2008:27). Altogether, this information is central to the establishment of a sound health and medicine approach in Africa which allows significant levels of interaction between medicine and humanities. Such interaction creates a platform for medical professionals (in the context of HIV and AIDS) to understand social practices and belief systems central to interpretation of illness and diseases, health-seeking behaviour and how the ultimate decision to utilise specific healing practices is arrived at.

## Note

1. The multi-faceted crisis has resulted in collapse of the economy, the health sector, water and service delivery and virtually all public services. A majority of the work force, rural and urban alike, faces deepening poverty, if not starvation. Hyperinflation has wiped out savings, while falling production and inability to pay for imports has caused major shortages of food, electricity, fuel and all basic goods. Most significant economic transactions are in dollars […] At the same time […] it is increasingly difficult to distribute essential anti-retrovirals and nutritional supplements. […] The rapidly deteriorating humanitarian situation, including […] the hunger of millions of the country's population, intensified discussion of the responsibility to protect the population from its government. (International Crisis Group, working to prevent conflict worldwide, 2008)
